# Antiparasitic Efficacy of Herbal Extracts and Active Compound Against *Gyrodactylus kobayashii* in *Carassius auratus*

**DOI:** 10.3389/fvets.2021.665072

**Published:** 2021-04-06

**Authors:** Shun Zhou, Jing Dong, Yongtao Liu, Qiuhong Yang, Ning Xu, Yibin Yang, Xiaohui Ai

**Affiliations:** ^1^Yangtze River Fisheries Research Institute, Chinese Academy of Fishery Sciences, Wuhan, China; ^2^Hu Bei Province Engineering and Technology Research Center of Aquatic Product Quality and Safety, Wuhan, China

**Keywords:** antiparasitic activity, herbal medicine, dioscin, *Gyrodactylus kobayashii*, *Dioscorea collettii* var. *hypoglauca*

## Abstract

*Gyrodactylus* spp. Nordmann, 1832 (Monogenea: Gyrodactylidae) are common ectoparasites of teleost fishes. Infection with these parasites can increase the mortality of fish and cause considerable economic losses in intensive aquaculture. To find an effective antiparasitic agent for the control of gyrodactylosis, antiparasitic efficacy of crude extracts of 36 herbal medicines was evaluated using a *Carassius auratus* (Cypriniformes, Cyprinidae)—*Gyrodactylus kobayashii* model. Among all tested medicines, methanol extract of *Dioscorea collettii* var. *hypoglauca* (Dioscoreales, Dioscoreaceae) was the most efficient, with an EC_50_ value of 4.17 mg/L. This extract showed 100% antiparasitic efficacy against *G. kobayashii* at 10 mg/L and had a therapeutic index (TI, LC_50_/EC_50_) of 5.26, which is higher than that of formaldehyde (TI = 4.58), a widely used parasiticide in aquaculture. Subsequently, the potential mechanism of antiparasitic activity of dioscin, an active compound isolated from *D. collettii* var. *hypoglauca* was investigated and the histopathological alterations in goldfish after exposure to dioscin were also studied. The *in vivo* trial indicated dioscin showed significant antiparasitic activity with a 24 h-EC_50_ value of 1.58 mg/L and it exhibited 100% antiparasitic efficacy at 0.6 mg/L. Also, *G. kobayashii* could be completely removed *in vivo* within 2 h at 0.6 mg/L dioscin. Whereas, mean survival time of this worm *in vitro* was 4.99 h, and some individuals even reached 12 h at the same concentration of dioscin. These results indicated that 0.6 mg/L of dioscin did not completely kill all worms within 2 h, but just temporarily remove the worms from goldfish. Scanning electron microscopy (SEM) analysis showed that most of the microvilli on the tegument surface of *G. kobayashii* dropped after exposure to dioscin. This might be one of the potential mechanisms of antiparasitic activity of dioscin against *G. kobayashii*. Furthermore, no severe histopathological alteration was observed after exposure to a high concentration of dioscin for a short time. Considering both effectiveness and safety, therapeutic baths with a high concentration of dioscin for a short time might be a more optimal choice for the treatment of gyrodactylosis in aquaculture.

## Introduction

*Gyrodactylus* spp. Nordmann, 1832 (Monogenea: Gyrodactylidae) are common flatworm ectoparasites of teleost fishes, which received an ample amount of scientific attention for their ability to cause a sharp drop in the production of salmonids and other freshwater fish (1, 2). They have a direct life-cycle and several generations occur within an individual parasite. New-born flatworms are capable of directly infecting the primary host (3, 4). These parasites can infect entire fish stocks and reach high infection intensities rapidly because of their high transmission rate and short generation time (5). Gyrodactylids attach to the host's epithelium through sclerotized hooks and feed on mucus and epithelial cells, resulting in damage to the fish tissue (2). The damage of the epidermis facilitates the invasion of secondary infections with other pathogens, which can increase the mortality of fish and then cause considerable economic losses due to epidemics in intensive aquaculture (2, 6).

To control gyrodactylosis in aquaculture, several chemical substances, including mebendazole, formalin, hydrogen peroxide and praziquantel, have been widely used (7–10). However, frequent use of these chemical substances results in the development of resistance to drugs, environmental contamination, as well as risks to human health (11, 12). It is thus necessary to find alternative and effective therapeutics to treat gyrodactylosis. Herbal medicines have been considered as alternative options due to their biodegradability and environmental friendliness (13). A previous study indicated that methanol extract of *Paris polyphylla* (Liliales, Melanthiaceae) is an effective alternative agent to control gyrodactylid infections, but its feasibility in the practical application has been largely restricted due to its high price (14). The saponins are believed to be the main active components of *P. polyphylla*, and previous studies and unpublished data also indicated that some saponins, such as gracillin, are effective against gyrodactylosis (14, 15). Saponins are structurally and biologically diverse phytochemicals that are widely occurring in many plants from the families Dioscoreaceae, Liliaceae, Solanaceae, Asclepiadaceae, etc. (16). Therefore, screening an effective and inexpensive agent from herbal medicines containing saponins may be a promising research direction.

The dried rhizome of *Dioscorea collettii* var. *hypoglauca* (Dioscoreales, Dioscoreaceae) is a traditional Chinese medicine widely used for the treatment of cervical carcinoma, carcinoma of the bladder and renal tumor (17). Previous researches indicated that ethanol extract of *D. collettii* var. *hypoglauca* and its active ingredients induced morphological abnormality of *Pyricularia oryzae* (Magnaporthales, Magnaporthaceae) and displayed cytotoxicity to cancer cells (18, 19). However, antiparasitic activity of *D. collettii* var. *hypoglauca* has not yet been reported up to now. Also, the analysis of the chemical constituents showed that the content of dioscin in the *D. collettii* var. *hypoglauca* was higher and dioscin has been proved to show good antiparasitic activity against *G. kobayashii* (14, 20). However, the antiparasitic mechanism of this compound has not been studied. *Solanum nigrum* (Solanales, Solanaceae) is a member of the Solanaceae family that has been widely used in traditional Chinese medicine (17). It has been found that *S. nigrum* contains steroid alkaloids, steroidal saponins and flavonoids, etc., which may be responsible for a broad spectrum of pharmacological activities (21). For example, Pestana et al. (22) found that acetone extract of *S. nigrum* showed 100% nematocidal activity against *Pratylenchus goodeyi* (Tylenchida, Pratylenchidae) after 23-h exposure. Also, crude aqueous extracts of *S. nigrum* displayed evident antiparasitic activity against the larva of *Fasciola hepatica* (Plagiorchiida, Fasciolidae) (23). However, to the best of our knowledge, antiparasitic activity of *S. nigrum* against fish monogeneans has not been reported so far.

To find an effective agent for the treatment of gyrodactylosis, in the current study, 36 herbal medicines were obtained and antiparasitic efficacy of their crude extracts was evaluated. Two kinds of herbal medicines (*D. collettii* var. *hypoglauca* and *S. nigrum*) with high antiparasitic activity were further fractionated with different polarity solvents and then investigated for their antiparasitic activity against *Gyrodactylus kobayashii* and acute toxicity against its host, goldfish (*Carassius auratus*). Also, to explore the potential mechanism of antiparasitic activity of dioscin, an active compound isolated from *D. collettii* var. *hypoglauca*, the *in vitro* and *in vivo* assays were conducted and the morphological alterations of *G. kobayashii* after exposure to dioscin were examined by scanning electron microscopy. Moreover, the histopathological alterations in goldfish after exposure to dioscin were investigated to explore the influence of dioscin on the experimental fish. These results might help understand the underlying antiparasitic mechanism of dioscin and facilitate its further exploitation and practical application in aquaculture.

## Materials and Methods

### Fish-Parasite Model

Healthy goldfish (mean body weight of 4.37 ± 0.58 g) were obtained from a local fish farm (Hanjin Ornamental Fish Farm, Wuhan, China) and then subjected to continuous baths with suitable concentrations of formalin solutions for 12 h at 48-h intervals to remove all ectoparasites after acclimation for 7 days. Ten goldfish were randomly selected for parasitological examination to ensure these fish were free of ectoparasites after a 30-day recovery. And then, *G. kobayashii*-infected goldfish maintained in the laboratory were mix-cultured with these ectoparasite-free goldfish for about 10 days at a ratio of 1:4 to obtain more infected goldfish. The goldfish-*G. kobayashii* model has been established and maintained in the laboratory referring to the methods reported in previous studies (24, 25).

### Preparation of Plant Materials and Chemicals

Thirty-six dried plant materials (Table 1) were purchased from a local Chinese pharmacy and then dried in an oven at 60°C for 48 h. These dry plant materials were then ground into powder with an electric grinder and freeze-dried at −45°C to completely remove water. The dry powder (30 g) of each plant material was extracted with 300 mL of anhydrous ethanol 3 times at 25°C; each extraction lasted for 48 h in a static state. Then the extracts were percolated through filter paper and concentrated under reduced pressure in a vacuum rotary evaporator (YRE2000E) at 50°C to obtain solidified crude extracts, which were then dissolved in dimethyl sulfoxide (DMSO) to get 0.5 g/mL (sample/solvent) stocking solutions. Dioscin was purchased from Shanghai Yuanye Biotech Co., Ltd. (Shanghai, China) and then dissolved in DMSO to get 2 mg/mL stocking solutions.

**Table 1:** Summary of tested plants used in the trial.

**Plants**	**Family**	**Plant part used**	**The highest AE (%)**	**CAE (mg/L)**	**CFD (mg/L)**
*Dioscorea collettii* var. *hypoglauca* (Palib.) S. J. Pei & C. T. Ting	Dioscoreaceae	Rhizome	100	15	25
*Dioscorea nipponica* Makino	Dioscoreaceae	Rhizome	100	35	35
*Solanum nigrum* Linn.	Solanaceae	Fruit	100	100	200
*Dioscorea spongiosa* J. Q. Xi, M. Mizuno et W. L. Zhao	Dioscoreaceae	Rhizome	100	250	400
*Cynanchum otophyllum* Schneid.	Asclepiadaceae	Root	100	800	1,000
*Dioscorea bulbifera* Linn.	Dioscoreaceae	Tuber	100	800	900
*Tupistra chinensis* Baker.	Liliaceae	Rhizome	100	800	1,000
*Smilax china* L.	Liliaceae	Rhizome	100	900	1,000
*Solanum americanum* Mill.	Solanaceae	Whole plant	98.16	1,000	1,000
*Reineckea carnea* (Andrews) Kunth	Liliaceae	Whole plant	97.82	1,000	>1,000
*Dioscorea zingiberensis* C. H. Wright	Dioscoreaceae	Rhizome	95.64	100	100
*Cynanchum bungei* Decne.	Asclepiadaceae	Root tuber	94.36	1,000	1,000
*Smilax glabra* Roxb.	Liliaceae	Rhizome	92.36	250	250
*Cynanchum stauntonii* (Decne.) Schltr. et Lévl.	Asclepiadaceae	Rhizome and root	90.24	1,000	1,000
*Periploca forrestii* Schltr.	Asclepiadaceae	Whole plant	82.27	1,000	>1,000
*Cynanchum atratum* Bunge	Asclepiadaceae	Rhizome and root	80.74	150	150
*Rohdea japonica* (Thunb.) Roth	Liliaceae	Rhizome and root	77.96	200	200
*Lilium brownii* F. E. Brown var. viridulum Baker	Liliaceae	Scale leaf	76.62	1,000	1,000
*Tribulus terrestris* L.	Zygophyllaceae	Fruit	76.53	1,000	>1,000
*Solanum lyratum* Thunb.	Solanaceae	Root and whole plant	73.96	1,000	1,000
*Polygonatum sibiricum* Red.	Liliaceae	Rhizome	69.44	800	800
*Fritillaria thunbergii* Miq.	Liliaceae	Bulb	66.92	1,000	>1,000
*Ophiopogon japonicus* (Linn. F.) Ker-Gawl.	Liliaceae	Root tuber	63.58	1,000	>1,000
*Solanum nigrum* Linn.	Solanaceae	Whole plant	50.63	1,000	1,000
*Trigonella foenum-graecum* L.	Leguminosae	Seed	44.28	1,000	1,000
*Trillium tschonoskii* Maxim.	Liliaceae	Rhizome and root	43.76	30	30
*Periploca calophylla* (Wight) Falc.	Asclepiadaceae	Stem	37.05	1,000	>1,000
*Curcuma longa* Linn.	Zingiberaceae	Rhizome	32.48	100	100
*Aspidistra elatior* Blume	Liliaceae	Rhizome	24.36	200	200
*Asparagus cochinchinensis* (Lour.) Merr.	Liliaceae	Root tuber	13.72	1,000	>1,000
*Hosta plantaginea* (Lam.) Aschers.	Liliaceae	Fruit	10.26	100	100
*Anemarrhena asphodeloides* Bunge	Liliaceae	Rhizome	8.64	300	300
*Marsdenia tenacissima* (Roxb.) Moon	Asclepiadaceae	Rattan stem	7.62	800	800
*Allium macrostemon* Bge.	Liliaceae	Bulb	0	900	900
*Dioscorea polystachya* Turcz.	Dioscoreaceae	Rhizome	0	1,000	>1,000
*Polygonatum odoratum* (Mill.) Druce	Liliaceae	Rhizome	0	1,000	>1,000

*AE, Antiparasitic efficacy against Gyrodactylus kobayashii in goldfish (Carassius auratus) of ethanol extracts; CAE, the concentration with the best AE; CFD, the concentration causing fish mortality*.

### *In vivo* Screening of 36 Plant Extracts for Antiparasitic Efficacy

Antiparasitic efficacy of these ethanol extracts against *G. kobayashii* was evaluated in 720 1-L tanks containing one infected goldfish and 0.5 L of dechlorinated water at 23.0 ± 0.6°C. A negative control group without drugs and a positive control group containing 0.2% DMSO were set up under the same conditions as the test groups. Formaldehyde, a widely used agent to treat gyrodactylosis, was also evaluated as a positive control agent with concentrations of 15, 20, 25, 30, and 35 mg/L. For ethanol extracts of 36 tested herbal medicines, the concentrations used in this trial ranged 15–100 mg/L for ethanol extracts of *D. collettii* var. *hypoglauca, Dioscorea nipponica, Dioscorea zingiberensis, Trillium tschonoskii, Curcuma longa* and *Hosta plantaginea*, and ranged from 100 to 300 mg/L for ethanol extracts of *S. nigrum, Dioscorea spongiosa, Smilax glabra, Cynanchum atratum, Rohdea japonica, Aspidistra elatior* and *Anemarrhena asphodeloides*. The concentrations of the remaining ethanol extracts ranged from 600 to 1,000 mg/L. All experiments were performed in ten replicates. Normally swimming goldfish with medium infection intensity (40–200 parasites/fish) were chosen for the *in vivo* assay. The experimental fish was anesthetized with 0.02% MS222 (tricaine methanesulfonate) and the number of *G. kobayashii* on the caudal fin was counted under a stereomicroscope at 0 and 48 h post-treatment. Antiparasitic efficacy was calculated according to the methods described in a previous study and the formula was as follows: E = (L–L_t_)/L × 100% for L > L_t_, E = 0 for L ≤ L_t_. In this formula, E is antiparasitic efficacy, L is the infection intensity of *G. kobayashii* before treatment, and L_t_ is the infection intensity after 48-h treatment (25–27).

**Figure 1:**
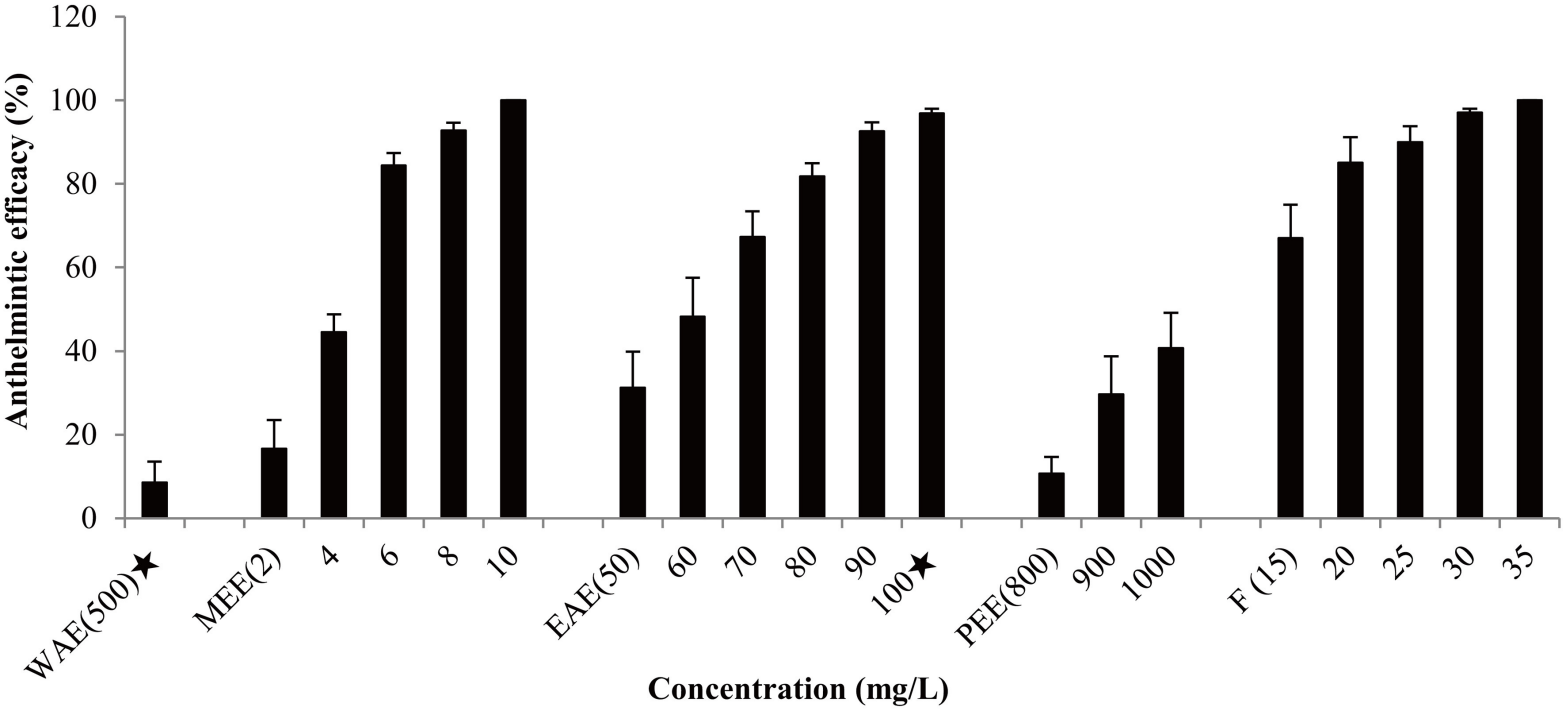
Antiparasitic efficacy of different extracts of *Dioscorea collettii* var. *hypoglauca* and formaldehyde against *Gyrodactylus kobayashii* after a 48-h treatment. WAE, water extract; MEE, methanol extract; EAE, ethyl acetate extract; PEE, petroleum ether extract; F, formaldehyde. A star indicates that fish death occurred.

**Figure 2:**
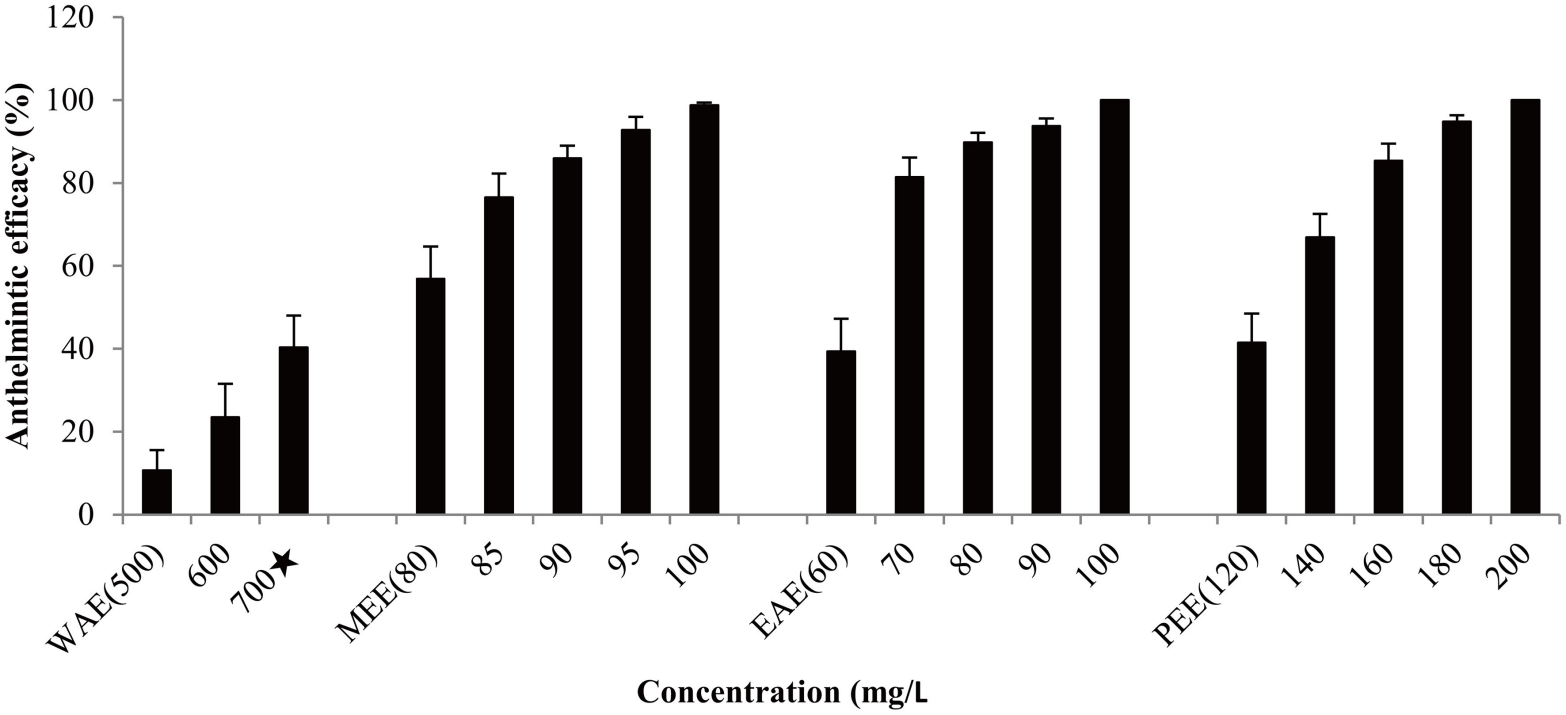
Antiparasitic efficacy of different extracts of *Solanum nigrum* against *Gyrodactylus kobayashii* after a 48-h treatment. WAE, water extract; MEE, methanol extract; EAE, ethyl acetate extract; PEE, petroleum ether extract. A star indicates that fish death occurred.

Based on effectiveness, safety and dosage, *D. collettii* var. *hypoglauca* and *S. nigrum* were selected for further extraction tests with different polarity solvents: water, methanol, ethyl acetate and petroleum ether. These extracts were obtained and then evaluated for their antiparasitic efficacy according to the afore-mentioned methods. For different polarity solvents extracts of *D. collettii* var. *hypoglauca* and *S. nigrum*, the concentrations used in the *in vivo* assay were presented in Figures 1, 2 and ranged from 2 to 200 mg/L for methanol and ethyl acetate extracts of these two plants and petroleum ether extract of *S. nigrum*. The concentrations of the remaining extracts ranged from 500 to 1,000 mg/L.

### Acute Toxicity Tests of Selected Extracts Against Goldfish

To evaluate the safety margin of different extracts of *D. collettii* var. *hypoglauca* and *S. nigrum*, acute toxicity tests against goldfish were conducted using an aqueous static bioassay method. A batch of healthy goldfish were randomly placed into 117 10-L aquariums (10 fish/aquarium) with 6 L of dechlorinated water and exposed to tested extracts for 48 h at 23.0 ± 0.6°C. The concentrations used in the acute toxicity tests ranged from 10 to 300 mg/L for methanol and ethyl acetate extracts of *D. collettii* var. *hypoglauca* and methanol extracts of *S. nigrum*. The concentrations of the remaining five extracts ranged from 400 to 1,000 mg/L (**Table 3**). A negative control group without drugs and a positive control group containing 0.2% DMSO were set up under the same conditions. Acute toxicity of formaldehyde (30, 40, 50, 60, and 70 mg/L) to goldfish was also evaluated. Three replicate aquariums were used for all treatment and control groups. Dead fish were recorded and immediately removed during the 48-h continuous exposure.

### *In vitro* Antiparasitic Efficacy of Dioscin Against *G. kobayashii*

Parasites were gently dislodged from the caudal fin of *G. kobayashii*-infected goldfish after anesthesia with MS-222 using fine insect pins under a stereomicroscope (28, 29). The individual parasite was randomly selected and transferred to a 24-well microtiter plate in 50 μL of dechlorinated water using a micropipette (a single worm per well). Each well was then added with 350 μL of dechlorinated water and cultured at 23.0 ± 0.6°C. After an hour of acclimatization, all wells were examined and the parasites that died or showed any abnormal behavior were excluded from the experiment. Then 100 μL of different concentrations of stock solutions were added to achieve the required concentrations (0.3, 0.4, 0.5, and 0.6 mg/L) and the time was defined as zero. A control group (a single parasite and 500 μL of dechlorinated water containing 0.06% DMSO in each well) was set up under the same conditions. Four parallel plates were used for all experimental groups. Subsequently, these parasites were observed every 1 or 2 h for their status until death occurred. The parasites that showed no response after a gentle stimulation by an insect pin were considered dead.

### *In vivo* Antiparasitic Efficacy of Dioscin Against *G. kobayashii*

The *in vivo* antiparasitic tests of dioscin were conducted as the above-described methods. Fifty infected goldfish were randomly placed into fifty tanks, each containing one infected goldfish and 0.5 L of dechlorinated water. Then the appropriate volume of stock solution was added into each tank to achieve the required concentrations of 0.6, 0.5, 0.4, 0.3, and 0 (control group, 0.06% DMSO) mg/L dioscin, respectively. The number of *G. kobayashii* on the caudal fin of the experimental fish was counted under a stereomicroscope at 0 and 24 h post-treatment and then antiparasitic efficacy was calculated.

Also, to evaluate the speed of parasite clearance of high concentrations of dioscin (0.4, 0.5, 0.6, 0.7, and 0.8 mg/L), the *in vivo* antiparasitic tests were further investigated as the above-described methods with minor modification. At the beginning of the experiment, each experimental fish was placed into a separate plastic box and the parasite load on the caudal fin was counted. Ten replicate plastic boxes were used for all groups (fifty experimental goldfish). Then experimental fish were exposed to various concentrations of dioscin for 4 h and the infection intensity of *G. kobayashii* on the caudal fin was examined every 1 h, and antiparasitic efficacy was calculated as aforementioned methods at each time point.

### Scanning Electron Microscopy

To determine whether dioscin caused morphological alterations to *G. kobayashii* tegument, SEM analysis of gyrodactylids samples was conducted following standard methods (30). In short, *G. kobayashii*-infected goldfish were exposed to 0.06% DMSO (control group) and 0.29 mg/L dioscin (24 h-EC_50_) for 12 h. Caudal fins with gyrodactylids were clipped and fixed in 2.5% glutaraldehyde solution for 24 h at 4°C, and then dehydrated by an alcohol gradient, dried with carbon dioxide and sputter-coated with gold particles. Finally, these processed samples were visualized using a scanning electron microscope (Hitachi SU8010, Tokyo, Japan).

### Histopathological Analysis After Exposures to Dioscin

To explore the influence of dioscin on the experimental fish, the histopathological alterations in goldfish after exposure to dioscin were investigated. Healthy goldfish were exposed to 0.06% DMSO (control group) and 0.6 mg/L dioscin (24 h-EC_100_) for 96 h, and the gill tissues from 10 fish (five fish per treatment) were sampled for histopathological analysis. To explore the influence of a high concentration of dioscin on the experimental fish, five healthy goldfish were subjected to therapeutic baths with 0.7 mg/L dioscin for 1 h and the gill tissues were also collected. All gill tissues were fixed in a 4% paraformaldehyde solution for 48 h and then dehydrated by an alcohol gradient, rinsed in xylene and embedded in paraffin wax. Finally, 5 μm sections were cut with a microtome and stained with hematoxylin and eosin, then the histological changes were evaluated using a Primo Star microscope (Carl Zeiss Microscopy GmbH, Jena, Germany).

### Statistical Analyses

The 50 and 90% effective concentrations for removing *G. kobayashii* (EC_50_ and EC_90_), and 50% and 90% lethal concentrations against goldfish (LC_50_ and LC_90_) with 95% confidence levels (CI) were calculated using the Probit method (31). The therapeutic index (TI) is defined as the ratio of LC_50_ to EC_50_ and high TI indicates a favorable safety (32). The survival rate of *G. kobayashii* exposed to different concentrations of dioscin was analyzed by Kaplan-Meier log-rank test (33). Statistical analysis was performed using SPSS 20.0 software.

## Results

### *In vivo* Screening of 36 Plant Extracts for Antiparasitic Efficacy

As Shown in Table 1, among the 36 herbal medicines, ethanol extracts of *D. collettii* var. *hypoglauca* and *S. nigrum* showed 100% antiparasitic efficacy at 15 and 100 mg/L after 48 h of exposure, respectively. Besides, ethanol extract of *D. nipponica* could completely remove gyrodactylids from goldfish at the concentration of 35 mg/L, but this concentration was lethal to goldfish. Ethanol extracts of five herbal medicines including *D. spongiosa, Cynanchum otophyllum, Dioscorea bulbifera, Tupistra chinensis*, and *Smilax china* also exhibited antiparasitic activity against *G. kobayashii* but at relatively high concentrations. The remaining ethanol extracts of herbal medicines showed varying degrees of antiparasitic efficacy ranging from 98.16% to 0.

Antiparasitic efficacy of different extracts of *D. collettii* var. *hypoglauca* and *S. nigrum* are illustrated in Figures 1, 2, and the results of the *in vivo* assay are summarized in Table 2. Among all tested extracts, methanol extract of *D. collettii* var. *hypoglauca* was the most efficient with 100% antiparasitic efficacy at a relatively low concentration of 10 mg/L. This extract had low EC_50_ and EC_90_ values of 4.17 and 7.04 mg/L after 48 h of exposure, respectively. Ethyl acetate extract of *D. collettii* var. *hypoglauca* also exhibited superior antiparasitic activity with an EC_50_ value of 60.6 mg/L and an EC_90_ value of 87.52 mg/L. Nevertheless, water and petroleum ether extracts of *D. collettii* var. *hypoglauca* showed weak antiparasitic activity even at the concentration of 500 or 1,000 mg/L (Figure 1 and Table 2). In the case of *S. nigrum*, methanol and ethyl acetate extracts showed 98.72 and 100% antiparasitic efficacy at the concentration of 100 mg/L, respectively (Figure 2 and Table 2). Water and petroleum ether extracts exhibited weak antiparasitic activity with relatively high EC_50_ values (more than 100 mg/L). In the two control groups, a significant rise in the gyrodactylids load in goldfish was observed, indicating that 0.2% DMSO and dechlorinated water showed no antiparasitic activity against *G. kobayashii*. As a positive control agent, formaldehyde showed 100% antiparasitic efficacy at 35 mg/L with EC_50_ and EC_90_ values of 10.5 and 23.4 mg/L, respectively (Figure 1 and Table 2).

**Table 2:** Antiparasitic efficacy (EC_50_ and EC_90_) of different extracts from *Dioscorea collettii* var. *hypoglauca* and *Solanum nigrum* against *Gyrodactylus kobayashii* after 48 h of exposure.

**Plants or agents**	**Extraction solvent**	**EC_**50**_ (mg/L)**	**95% CI**	**EC_**90**_ (mg/L)**	**95% CI**
*D. collettii* var. *hypoglauca*	Water	>500	–	–	–
	Methanol	4.17	3.82–4.5	7.04	6.6–7.61
	Ethyl acetate	60.6	57.44–63.27	87.52	83.99–92.1
	Petroleum ether	>1,000	–	–	–
*S. nigrum*	Water	748.26	701.44–847	1005.43	889.99–1275.11
	Methanol	77.9	74.49–80.13	91.85	90.09–94.3
	Ethyl acetate	61.46	39.76–68.68	80.62	73.41–102.14
	Petroleum ether	126.99	120.66–131.94	166.14	160.67–173.43
Formaldehyde	–	10.5	6.1–13.1	23.4	21.8–25.6

EC_50_, effective concentration with 50% Antiparasitic efficacy; EC_90_, effective concentration with 90% Antiparasitic efficacy; 95% CI, 95% confidence interval; –, not calculated.

### Acute Toxicity Tests of Selected Extracts Against Goldfish

The results of acute toxicity tests of different extracts are presented in Table 3. Among all tested extracts, only two had higher TI values than that of a widely used parasiticide-formaldehyde (TI value of 4.58), namely methanol extract of *D. collettii* var. *hypoglauca* (TI value of 5.26) and ethyl acetate extract of *S. nigrum* (TI value of 10.99). Petroleum ether extracts of these two herbal medicines exhibited low acute toxicity to goldfish; only one or two dead fish were observed at the concentration of 1,000 mg/L. The remaining extracts had lower TI values than formaldehyde. In the control groups with no drug and DMSO, no deaths were observed, suggesting that the impact of DMSO on goldfish should be negligible.

**Table 3:** Acute toxicity of different extracts from *Dioscorea collettii* var. *hypoglauca* and *Solanum nigrum* against goldfish after 48 h of exposure.

**Plants or agents**	**Extraction solvent**	**LC_**50**_ (mg/L)**	**95% CI**	**LC_**90**_ (mg/L)**	**95% CI**	**TI (LC_**50**_/EC_**50**_)**
*D. collettii* var. *hypoglauca*	Water	778.48	723.24–838.59	1130.72	1026.27–1329.12	–
	Methanol	21.93	20.15–23.84	31.53	28.74–36.19	5.26
	Ethyl acetate	194.33	179.37–209.25	267.01	247.05–297.78	3.21
	Petroleum ether	>1,000	–	–	–	–
*S. nigrum*	Water	905.98	869.82–951.74	1088.35	1023.65–215.53	1.21
	Methanol	160.79	152.66–169.02	205.26	192.73–226.86	2.06
	Ethyl acetate	675.37	640.05–716.95	864.98	805.2–967.84	10.99
	Petroleum ether	>1,000	–	–	–	–
Formaldehyde	–	48.1	44.5–51.6	61.5	57.1–68.9	4.58

*LC_50_, 50% lethal concentration; LC_90_, 90% lethal concentration; 95% CI, 95% confidence interval; TI, therapeutic index; –, not calculated*.

### *In vitro* and *in vivo* Antiparasitic Efficacy of Dioscin Against *G. kobayashii*

**Figure 3:**
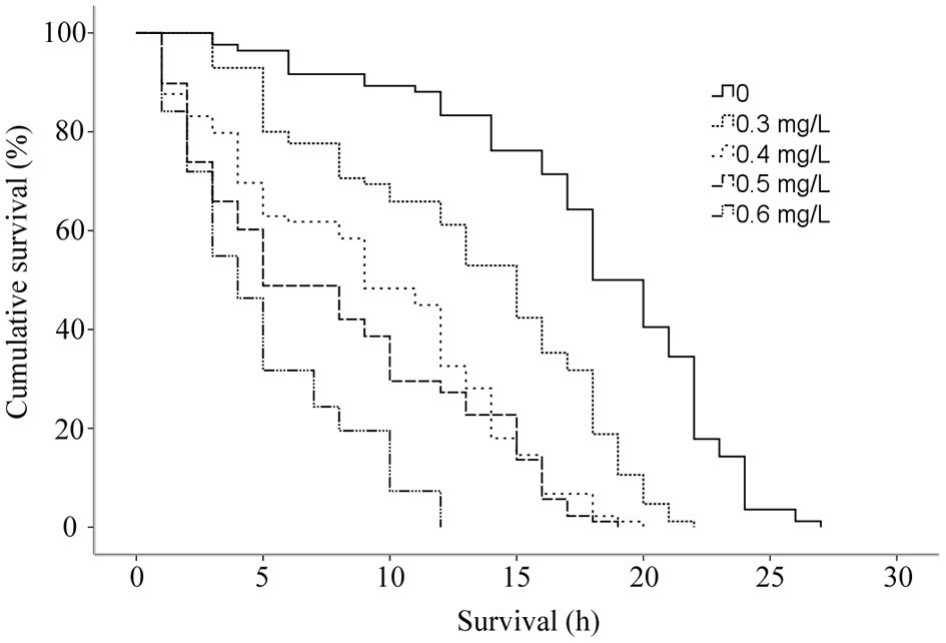
Cumulative survival (%) *in vitro* of *Gyrodactylus kobayashii* at different concentrations of dioscin.

The cumulative survival of *G. kobayashii in vitro* decreased with increasing concentrations of dioscin (Figure 3), and a significant difference in the survival curves at different concentrations of dioscin was observed (*P* < 0.01). Mean survival time of *G. kobayashii in vitro* decreased in a dose-dependent manner and was reduced from 18.02 h in the control group to 4.99 h at the concentration of 0.6 mg/L dioscin (Table 4). At the high concentrations of dioscin (0.5 and 0.6 mg/L), the rapid and irregular twisting movements of the worms were observed just after exposure to dioscin, and the skin of the worms shrank and turned black within 1 h.

**Table 4:** *In vitro* effects of dioscin on *Gyrodactylus kobayashii*.

**Agents**	**Concentrations (mg/L)**	**Sample sizes**	**Mean survival time (h)**	**Standard error**	**Range of survival time (h)**
Dioscin	0	84	18.02	0.61	3–27
	0.3	85	13.05	0.62	3–22
	0.4	89	9.17	0.51	1–20
	0.5	88	7.72	0.6	1–19
	0.6	82	4.99	0.38	1–12

**Figure 4:**
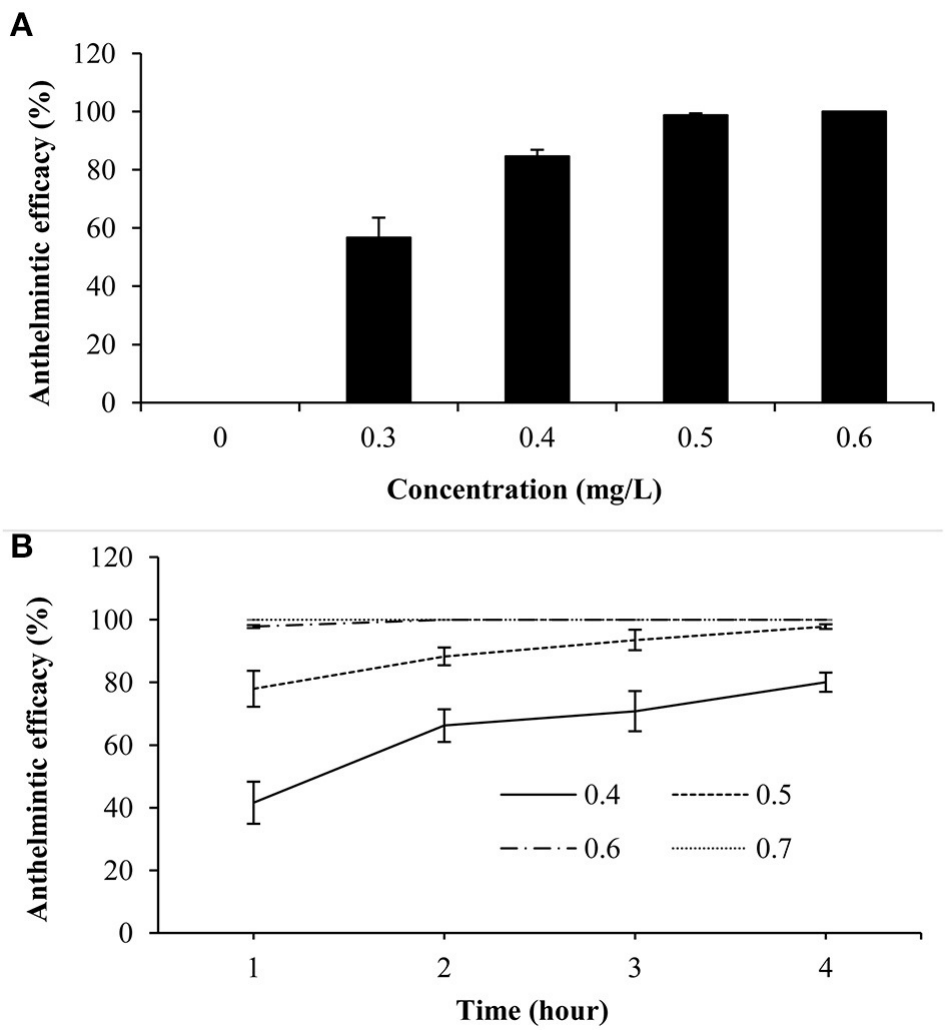
Antiparasitic efficacy of dioscin against *Gyrodactylus kobayashii* after 24-h treatment *in vivo* **(A)**; the effect of different concentrations of dioscin on the survival time of *Gyrodactylus kobayashii in vivo* **(B)**.

*In vivo* trial indicated that dioscin showed significant antiparasitic activity, with a 24 h-EC_50_ value of 0.29 mg/L, and it exhibited 100% antiparasitic efficacy against *G. kobayashii* at 0.6 mg/L (Figure 4A). The therapeutic baths with 0.5 mg/L of dioscin for 4 h could decrease 97.82% parasite loads in goldfish. Furthermore, all gyrodactylids could be completely eliminated from goldfish within 2 h at a concentration of 0.6 mg/L dioscin, and within 1 h at a concentration of 0.7 mg/L dioscin (Figure 4B).

### Scanning Electron Microscopy

**Figure 5:**
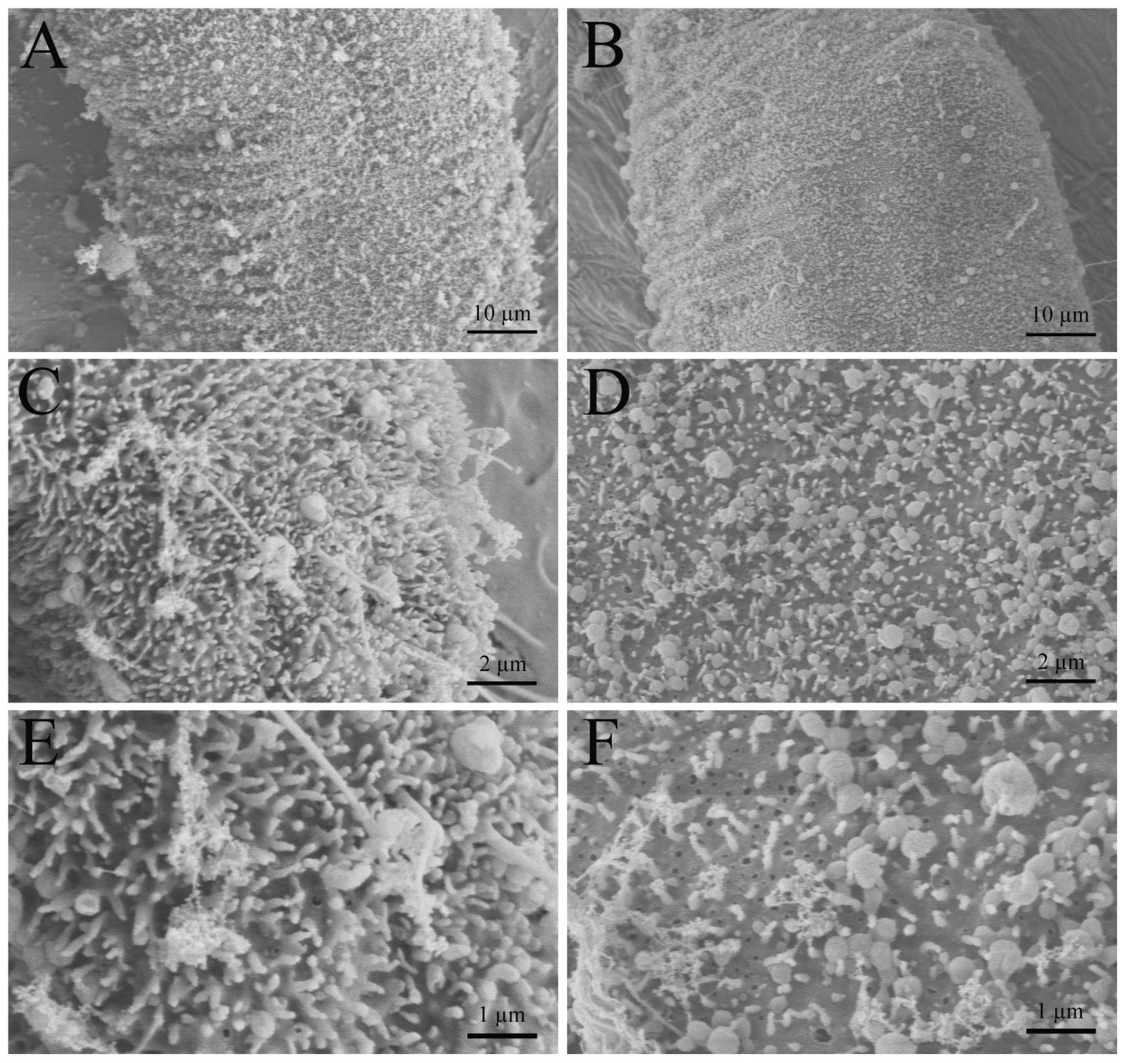
Scanning electron microscopy (SEM) of *Gyrodactylus kobayashii* exposed to dioscin. Control group: worms exposed to 0.06% DMSO for 12 h **(A,C,E)**, treatment group: worms exposed to 0.3 mg/L dioscin for 12 h **(B,D,F)**.

The morphological alterations of *G. kobayashii* tegument after exposure to dioscin observed by SEM are shown in Figure 5. *G. kobayashii* exposed to 0.06% DMSO (control group) displayed a defined body shape and dense microvilli on the tegument surface (Figures 5A,C,E). In contrast, parasites treated with 0.29 mg/L dioscin displayed substantial tegumental damage and most of the microvilli on the tegument surface dropped (Figures 5B,D,F).

### Histopathological Analysis After Exposures to Dioscin

**Figure 6:**
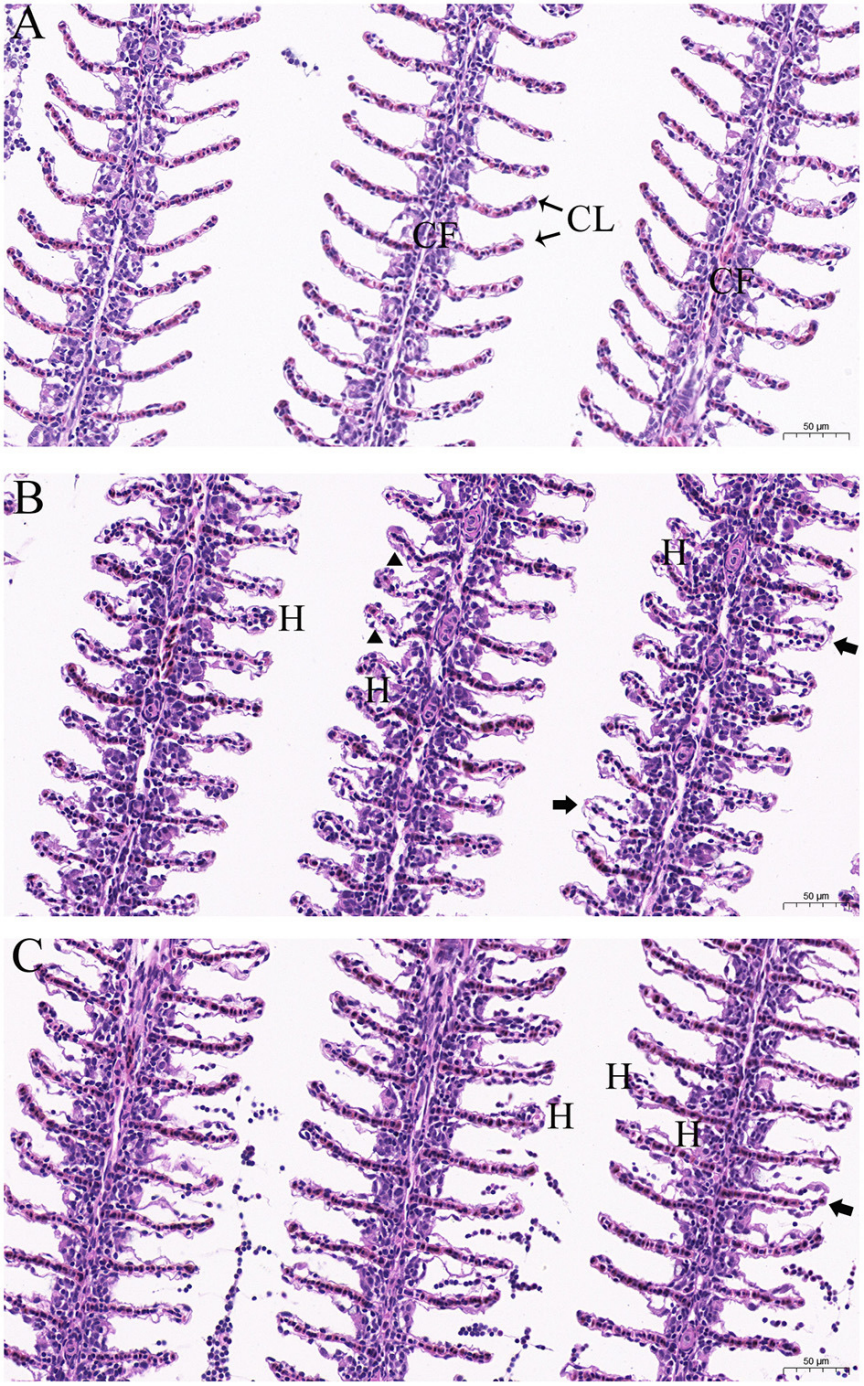
Histopathological characteristics in the gills of goldfish (*Carassius auratus*) after exposure to different concentrations of dioscin. **(A)** goldfish gills exposed to 0.06% DMSO for 96 h (control group), gill filament (GF) and gill lamellae (GL); **(B)** goldfish gills exposed to 0.6 mg/L dioscin for 96 h, hyperplasia (H), curling and abnormal elongation of the secondary lamellae (triangles) and detachment of lamellar epithelium (arrows); **(C)** goldfish gills exposed to 0.7 mg/L dioscin for 1 h, hyperplasia (H) and detachment of lamellar epithelium (arrows).

The structural details of the gill tissues of experimental goldfish are shown in Figure 6. In the control group, a normal morphological structure with each gill filament supporting a series of secondary gill lamellae was observed (Figure 6A). The gills of goldfish in the treatment groups showed an intact structural organization but with histopathological changes, including hyperplasia, detachment of lamellar epithelium, edema and curling and abnormal elongation of the secondary lamellae (Figures 6B,C).

## Discussions

In the current study, 36 kinds of herbal medicines containing saponins were obtained and investigated for the *in vivo* antiparasitic activity against *G. kobayashii* in goldfish. Based on the screening results, there was a considerable difference in the antiparasitic activity of these herbal medicines, despite all of them containing saponins. For example, ethanol extracts of *D. collettii* var. *hypoglauca, D. nipponica*, and *S. nigrum* showed 100% antiparasitic efficacy at relatively low concentrations, while ethanol extracts of *Marsdenia tenacissima, Allium macrostemon, Dioscorea polystachya*, and *Polygonatum odoratum* exhibited weak or no antiparasitic activity even at the concentration of 1,000 mg/L. Even for more closely related herbal medicines, such as *D. collettii* var. *hypoglauca* and *D. spongiosa*, slight changes in the saponin content might lead to a great difference in antiparasitic activity (34). The difference in the antiparasitic activity might thus be due to the varying contents of saponins in different herbal medicines. Besides, ethanol extracts of *D. spongiosa, C. otophyllum, D. bulbifera, T. chinensis*, and *S. china* also exhibited 100% antiparasitic activity against *G. kobayashii* but at relatively high concentrations. These results indicated that some active ingredients with antiparasitic activity exist in these extracts and need to be further isolated.

Based on effectiveness, safety and dosage, *D. collettii* var. *hypoglauca* and *S*. *nigrum* were selected for further extraction with different polarity solvents and then evaluated for their antiparasitic efficacy. Among all tested extracts, methanol extract of *D. collettii* var. *hypoglauca* was the most efficient, with EC_50_ and EC_90_ values of 4.17 and 7.04 mg/L, respectively. This extract exhibited 100% antiparasitic efficacy against *G. kobayashii* at a concentration of 10 mg/L, which is lower than that of methanol extract of *P. polyphylla*, an effective alternative agent to control gyrodactylids infections identified in a previous study (14). To our knowledge, the economic cost is an important determinant of the development of natural antiparasitic agents. The market price of *D. collettii* var. *hypoglauca* is far lower than that of *P. polyphylla*, which would increase the feasibility of the practical application of this herbal medicine. Besides, methanol extract of *D. collettii* var. *hypoglauca* had a TI value of 5.26, which is higher than that of formaldehyde (TI = 4.58), a widely used parasiticide in aquaculture. These results indicated that methanol extract of *D. collettii* var. *hypoglauca* was superior to formaldehyde in terms of antiparasitic efficacy and safety and thus might have the potential to be a novel antiparasitic agent to treat gyrodactylosis.

Besides, the *in vivo* trial indicated that the worms could be completely removed within 2 h at a concentration of 0.6 mg/L. Surprisingly, mean survival time of *G. kobayashii in vitro* was 4.99 h, and some individuals even reached 12 h after exposure to 0.6 mg/L dioscin. These results indicated that time to death of *G. kobayashii* was longer than time to detachment from host after exposure to dioscin, and 0.6 mg/L of dioscin did not completely kill all worms within 2 h, but just temporarily remove the worms from goldfish. A similar phenomenon was observed for praziquantel and mebendazole, which caused detachment of the parasites before full death (35). Also, the *in vitro* observation showed that exposure to a high concentration of dioscin caused rapid and irregular twisting movements of the parasites, which might be one of the reasons why the parasites rapidly shed from goldfish. Similarly, the worms treated with garlic extract also appeared to contract and twitch violently (36). These results indicated they may have similar modes of action on gyrodactylids, although their antiparasitic mechanisms are unclear. It was worth noting that detached gyrodactylids can survive *in vitro* for several hours and reinfect when they come into contact with a new host (37). These results would suggest that dioscin should be thus mixed with other antiparasitic drugs in practical applications to avoid the reinfection of the hosts by the detached parasites. Besides, the tegument integrity is critical to the survival of gyrodactylids (2). SEM analysis showed that most of the microvilli on the tegument surface dropped and obvious tegumental damage was observed after exposure to dioscin. This might be one of the potential mechanisms of antiparasitic activity of dioscin against *G. kobayashii*.

The therapeutic bath has been one of the most frequently used methods to treat gyrodactylosis in aquaculture and baths with high concentrations of agents inevitably cause adverse effects to fish (7). In teleost fishes, gills are the main respiratory organ and surrounded by agents in the therapeutic baths. The histopathological alterations in goldfish gills were thus investigated to explore the influence of dioscin on the experimental fish. Histopathological analysis indicated an intact structural organization with no severe histopathological alteration in the gills of goldfish after exposure to 0.6 mg/L dioscin for 96 h or 0.7 mg/L dioscin for 1 h. Also, the *in vivo* trial indicated gyrodactylids could be completely removed from goldfish within 2 h at a concentration of 0.6 mg/L dioscin, and within 1 h at a concentration of 0.7 mg/L dioscin. Generally, the adverse effects of chemotherapeutic agents on fish are dose and time-dependent, short bath time is thus beneficial to host. Considering both effectiveness and safety, the therapeutic baths with a high concentration of dioscin for a short time (the baths with 0.7 mg/L for 1 h) might thus be a more optimal choice for the treatment of gyrodactylosis in aquaculture.

In the screening experiment, ethanol extract of the fruit of *S. nigrum* displayed 100% antiparasitic efficacy at 100 mg/L, whereas ethanol extract of the whole plant of *S. nigrum* showed weak antiparasitic activity of 50.63% even at the concentration of 1,000 mg/L, which is lethal to goldfish. Glycoalkaloids, such as solamargine and solasonine, are believed to be the main active components of *S. nigrum*. The content of solasodine, the aglycone of the above-mentioned glycoalkaloids, is much higher in the fruit of *S. nigrum* than in the rest of the plant, which might be the underlying reason for the high antiparasitic activity of *S. nigrum* fruit extract (21, 38, 39). Also, ethyl acetate extract of *S. nigrum* fruit exhibited 100% antiparasitic activity at 100 mg/L with a TI value of 10.99, which is higher than that of formaldehyde. These results demonstrated that ethyl acetate extract of *S. nigrum* was safer than formaldehyde and exhibited considerable potential as an antiparasitic agent for the management of gyrodactylids infections.

In summary, this study demonstrated the potential of *D. collettii* var. *hypoglauca* and *S. nigrum* in the treatment of gyrodactylids infection in goldfish. Besides, dioscin showed significant antiparasitic activity and the therapeutic baths with a high concentration of dioscin for a short time might prove to be an effective management strategy in the control of gyrodactylosis in aquaculture. Moreover, these results further support the use of herbal medicines as promising sources of natural antiparasitic agents. Nonetheless, field studies are required to assess antiparasitic efficacy of extracts or active compounds under natural conditions.

## Data Availability Statement

The raw data supporting the conclusions of this article will be made available by the authors, without undue reservation.

## Ethics Statement

All animal experiments were approved and conducted in compliance with the experimental practices and standards developed by the Animal Welfare and Research Ethics Committee of Yangtze River Fisheries Research Institute (YFI2020zhoushun001). The animals used in this study were derived from commercial sources, and the owners' consent was not required. All surviving fish continued to be cultured in the laboratory in accordance with standard breeding procedures.

## Author Contributions

The design of the experiment was done by SZ and XA. SZ, JD, and YL: methodology. QY, NX, and YY: data collection and analysis. SZ and XA: writing of the manuscript. All authors have read and approved the manuscript.

## Conflict of Interest

The authors declare that the research was conducted in the absence of any commercial or financial relationships that could be construed as a potential conflict of interest.
